# Immediate liposuction could shorten the time for endoscopic axillary lymphadenectomy in breast cancer patients

**DOI:** 10.1186/s12957-017-1106-7

**Published:** 2017-01-31

**Authors:** Fujun Shi, Zonghai Huang, Jinlong Yu, Pusheng Zhang, Jianwen Deng, Linhan Zou, Cheng Zhang, Yunfeng Luo

**Affiliations:** Department of General Surgery, Zhujiang Hospital of Southern Medical University, 253, Gongye Dadao Zhong, Haizhu District, Guangzhou, China

**Keywords:** Liposuction, Endoscopic, Axillary lymphadenectomy breast cancer

## Abstract

**Background:**

Endoscopic axillary lymphadenectomy (EALND) was introduced to clinical work to reduce side effects of conventional axillary lymphadenectomy, while the lipolysis and liposuction of EALND made the process consume more time. The aim of the study was to determine whether immediate liposuction after tumescent solution injection to the axilla could shorten the total time of EALND.

**Methods:**

Fifty-nine patients were enrolled in the study, 30 of them received EALND with traditional liposuction method (TLM), and the rest 29 patients received EALND with immediate liposuction method (ILM). The operation time, cosmetic result, drainage amount, and hospitalization time of the two groups were compared.

**Results:**

The median EALND operation time of TLM group and ILM group were 68 and 46 min, respectively, the difference was significant (*P* < 0.05); the median cosmetic results of the two groups were 6.6 and 6.4, respectively; the median drainage amount of the two groups were 366 and 385 ml, respectively; the hospitalization time of the two groups were 15 and 16 days, respectively. For the last three measures, no significant difference was confirmed (*P* > 0.05).

**Conclusions:**

Our work suggests immediate liposuction could shorten the endoscopic axillary lymphadenectomy process, and this method would not compromise the operation results. However, due to the limitations of the research, more work needs to be done to prove the availability and feasibility of immediate liposuction.

## Background

Breast cancer threatens the health and life quality of women seriously all around the world. Lumpectomy, quadrantectomy, or mastectomy would be chosen to remove the tumor itself, axillary lymphadenectomy is carried out to decrease the local recurrence rate, help to define the pathological stage of the disease, and thus determine future therapy for these patients. Conventional axillary lymphadenectomy (CALND) was once widely used; however, about 80% of the patients who received the procedure would suffer from postoperative complication such as swelling, paresthesia, reduced arm mobility, lymphedema, seroma, and postoperative pain [1], although in recent years, CALND became less aggressive and complications such as paresthesia could still be as high as 53% [2]. Many researchers tried to find new surgical approaches which could reduce the inherent limitations of CALND complications, so as to better preserve arm function. Endoscopic axillary lymphadenectomy (EALND) was introduced to clinical work [3–5] and was proved to be able to reduce the abovementioned complications [6]. In the procedure, first lipolysis liquid would be injected into the axillary fossa, then about 15–20 min would be required to start liposuction [3, 5, 7], and EALND would be followed. However, as pointed out by Venkataram, when used for aesthetic purpose, liposuction might be carried out right after the tumescent liquid has been injected [8], but whether this might work for breast cancer surgery remains unclear. In this study, we tried to remove the waiting time so as to shorten the total operation time, and also, we want to find out whether this change would compromise the operation results.

## Methods

### Patients and groups

From May 2013 to October 2015, 59 female patients with invasive breast cancer were enrolled in the study.

All tumors were evaluated preoperatively using mammography and ultrasonography and diagnosed as invasive breast cancer pathologically through preoperative biopsy. The inclusion criteria were as follows: (1) all tumors are operable (M0), (2) maximum tumor diameter ≤50 mm, (3) with clinically palpable axillary lymph node, (4) no neoadjuvant chemotherapy has ever been applied, and (5) no serious diseases of other systems. All patients were informed about the details of the study, then they were assigned to the traditional liposuction method (TLM) group, *n* = 30) or immediate liposuction method (ILM) group, *n* = 29) by convenient sampling (with the help of random number table). The surgeons would not be informed about the group of the patient until the beginning of the operation, and for the patients, they would be informed the day postoperation.

### Surgical procedures

#### Preoperative preparation

This included marking the location of the tumor, the range of resection, and the border of the muscles in the operation field.

### Position and carbon nanoparticle injection

After general anesthesia with tracheal intubation has been applied, the patient was placed in the supine position with the diseased side raised by 30°, the affected breast would be placed near the edge of the operation table. The ipsilateral arm was abducted to 90°, and the forearm was hung along a frame which was fixed to the operation bed. After disinfection and draping were done, 50 mg of carbon nanoparticles (Lummy Pharmaceutical Limited, Chongqing, China) was injected subcutaneously in the areola in multiple points.

### Lipolysis liquid injection and liposuction

A 10-mm incision was made along the midaxillary line at the level of the lower margin of the breast (also used as the inspection trocar incision for the next step), about 400 ml of lipolysis liquid (250 ml of normal saline, 250 ml of sterile water, 30 ml of 5% NaHCO_3_, 20 ml of 2% lidocaine, and 1 ml of 0.1% adrenaline mixed to form a total volume of 551 ml) was injected into the axilla at superficial and deeper levels. Then, the axillary adipose tissue was aspirated with cannulae through the inspection incision immediately (ILM group) or started until 20 min later (TLM group).

### Endoscopic axillary lymphadenectomy

After liposuction, a 10-mm trocar was inserted into the liposuction hole. Through this trocar, medical CO_2_ gas was infused into the axilla to a maximum of 8 mmHg of pressure to establish the working space. Finally, two additional 5-mm trocars were placed in the anterior axillary line at the nipple level (first operating incision) and the posterior axillary line at the nipple level (second operating incision). The cobweb-like lymphatic and lymph nodes attached to the blood vessels were severed and peeled with an ultrasonic scalpel. Axillary level I and II lymph nodes were dissected, then axilla was washed with distilled water added with 5-Fu, a drainage tube would be inserted into the axilla through the inspection incision and fixed to the skin with suture after the EALND operation was finished. The lymph nodes and axillary tissues were sent for further pathological examination.

### Quadrantectomy or mastectomy

Based on the condition of the tumor, lumpectomy or mastectomy would be carried out after EALND with the open method; since our focus is on the *lipolysis liquid injection and liposuction* part, so the details of lumpectomy or mastectomy was omitted here.

### Postoperation management

After surgery, a pressure dressing was applied over the treatment site and kept in place for 72 h. The drainage tube was removed till the drainage amount was lower than 20 ml/day. Five days later, patients would accept clinical and ultrasonography assessment for the presence of seroma, and any residual liquid would be drawn out with a syringe.

### Measurement of the operation

The primary measures of the study were as follows:

1. Operation time: in our study, the operation time was composed of several parts, i.e., the lipolysis liquid injection time, the waiting time (20 min, only for TLM group), the liposuction time, and endoscopic axillary lymphadenectomy time. We used two time points, the first was the time point to make the 10-mm incision, the second was the time point at which the drainage tube has been fixed to the skin, by this way, we calculated and recorded the “operation time”; and it should be emphasized here that the time for quadrantectomy or mastectomy was not counted or added into the “operation time”
2. Cosmetic result: the patients enrolled in the study were asked to judge the cosmetic result with visual analogue scale (VAS) method
3. Drainage amount: the total postoperation drainage amount was calculated by the adding up daily drainage amount, which was recorded by the nurses
4. Hospitalization time: the hospitalization time of each patient enrolled in the study was recorded based on their medical records

Statistical analysis was performed on the patients who completed the postoperative evaluation using SPSS for Windows (Version 13.0; SPSS, Inc., Chicago, IL). Results were expressed as medians (ranges). Differences were compared with Mann-Whitney *U* test. *P* value <0.05 was considered statistically significant. The analyzer was blind to the grouping, data were transferred to him with the designation group 1 and group 2 as he carried out statistical analysis.

## Results

Altogether, 59 patients were enrolled into the study. The age, tumor size, histological types of the tumor, and lymph node removed in ILM group and TLM group are listed in Table 1 (patients’ characteristics); no significant differences were found between the two groups.

**Table 1:** Patients’ characteristics

	ILM group	TLM group
Age (years)	56 (33–66)	48 (30–63)
Tumor size (mm)	17 (9–48)	23 (7–43)
Histological type		
Invasive ductal carcinoma	27	30
Invasive lobular carcinoma	1	0
Mucinous carcinoma	1	0
Lymph node removed	19 (9–51)	17 (11–43)

We finished EALND smoothly for all the patients (both of the TLM group and ILM group); none of them was transferred to conventional axillary lymphadenectomy due to uncontrolled bleeding.

**Fig. 1:**
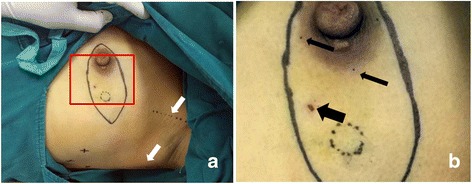
The photos of the operation process for ILM group. **a** Prior the surgery, *indicating lines* were drawn in the operation field. *Dotted direct lines* indicated the border of musculi pectoralis major (the *upper line, upper whiter arrow*) and latissimus dorsi (the *lower line, lower white arrow*), the *dotted circle* indicated the border of the tumor, “*X*” indicated the targeted positions for the three trocars, and the *continuous spindle-shaped line* indicated the targeted incision route for mastectomy. **b** Enlargement of the *inset* (*red rectangle*) in Fig. 1a. The *thick black arrow* indicated the point for the fine needle biopsy, the *dotted circle* indicates the border of the tumor, and the *thin black arrows* pointed to the injection site for carbon nanoparticles

**Fig. 2:**
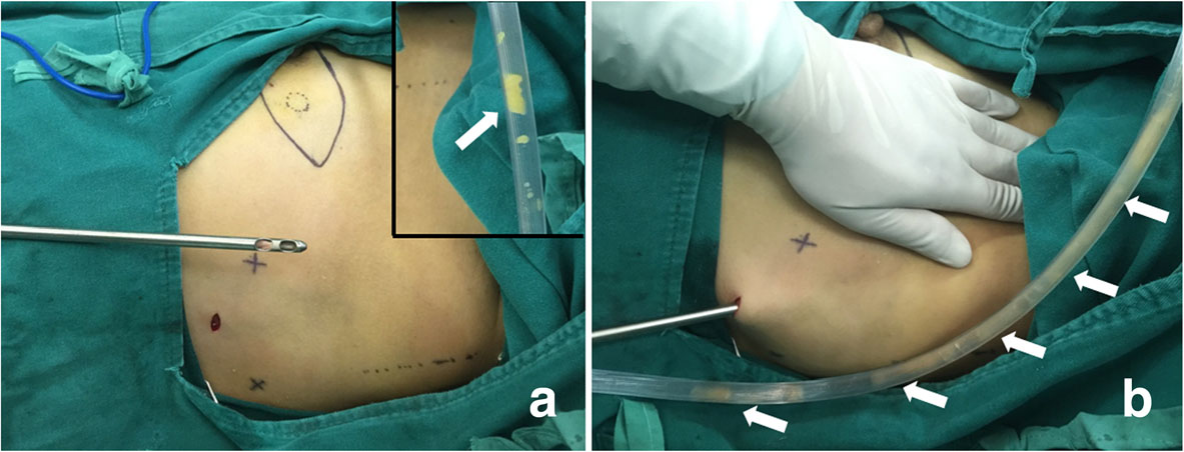
The photos of the operation process for ILM group. **a** A 10-mm incision was made to insert the needle so as to inject the tumescent fluid. In the first few minutes of liposuction, many adipose tissue particles were withdrawn into the tube (*white arrow in the inset*). **b** In the later part of the liposuction, adipose tissue particles became fewer, and oil-like liquid became more and were seen inside the tube (*white arrows*)

**Fig. 3:**
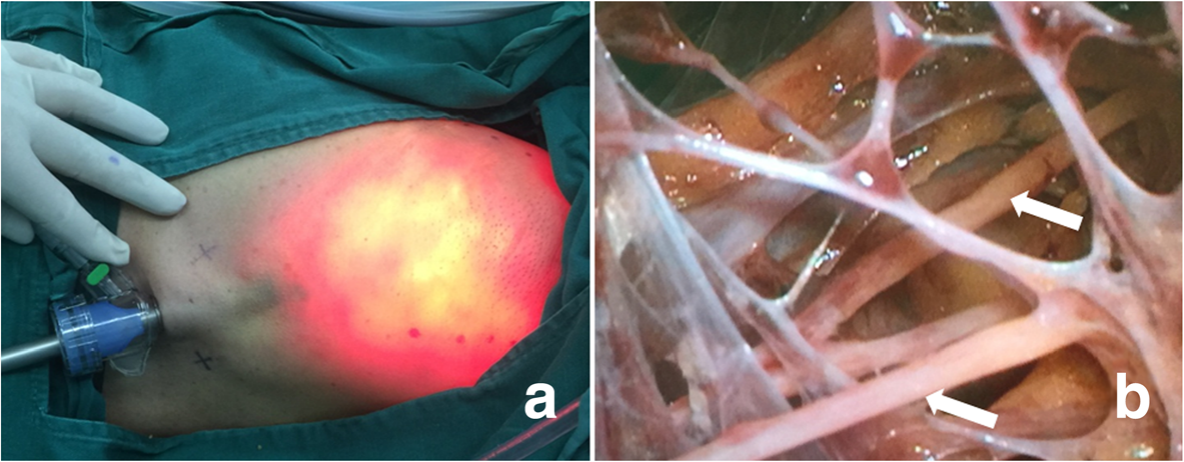
The photos of the operation process. **a** After liposuction, CO_2_ gas was injected into the axillary space and the lens was inserted into the 10-mm trocar. As indicated by the *red light*, a space has been successfully set up. **b** With the help of the lens, the nerves (*white arrows*) and fibrous tissue could be clearly located, and almost no adipose tissue was visible

**Fig. 4:**
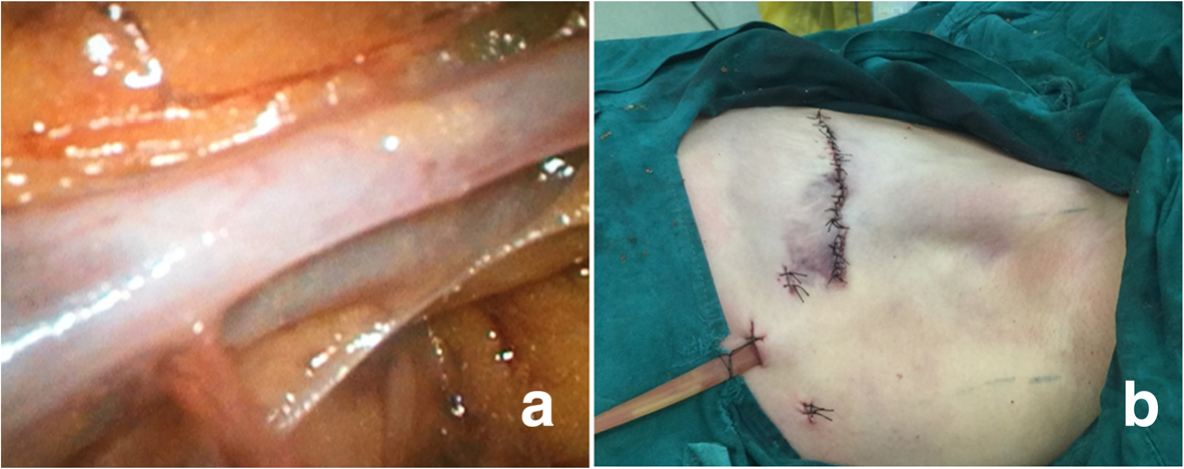
The photos of the operation process. **a** After liposuction and with the help of the lens, the axillary blood vessels could be easily observed. **b** After endoscopic axillary lymphadenectomy, a drainage tube would be inserted into the 10-mm trocar incision and fixed to the skin, the rest 2 incisions were closed up with suture

First, necessary markings were drawn in the operation area (Fig. 1a) and carbon nanoparticles were injected (Fig. 1b, thin black arrows). After lipolysis liquid was injected to the axilla, the axillary adipose tissue was drawn out by the cannulae connected with negative pressure aspiration (Fig. 2a, b, white arrows) during ILM procedure (for TLM procedure, the results were similar and figures were not shown). After liposuction, a 10-mm trocar was inserted into the incision and medical carbon dioxide gas was injected into the cavity. The desired operation axillary space was successfully established which could be confirmed by the lens lighting of endoscopic system (Fig. 3a); after the lens was inserted into the cavity via the trocar, the axillary nerves (Fig. 3b, arrows) and axillary blood vessels (Fig. 4a) could be easily recognized. After EALND was finished, a drainage tube was inserted into the inspection incision and fixed to the skin by the suture (Fig. 4b).

**Fig. 5:**
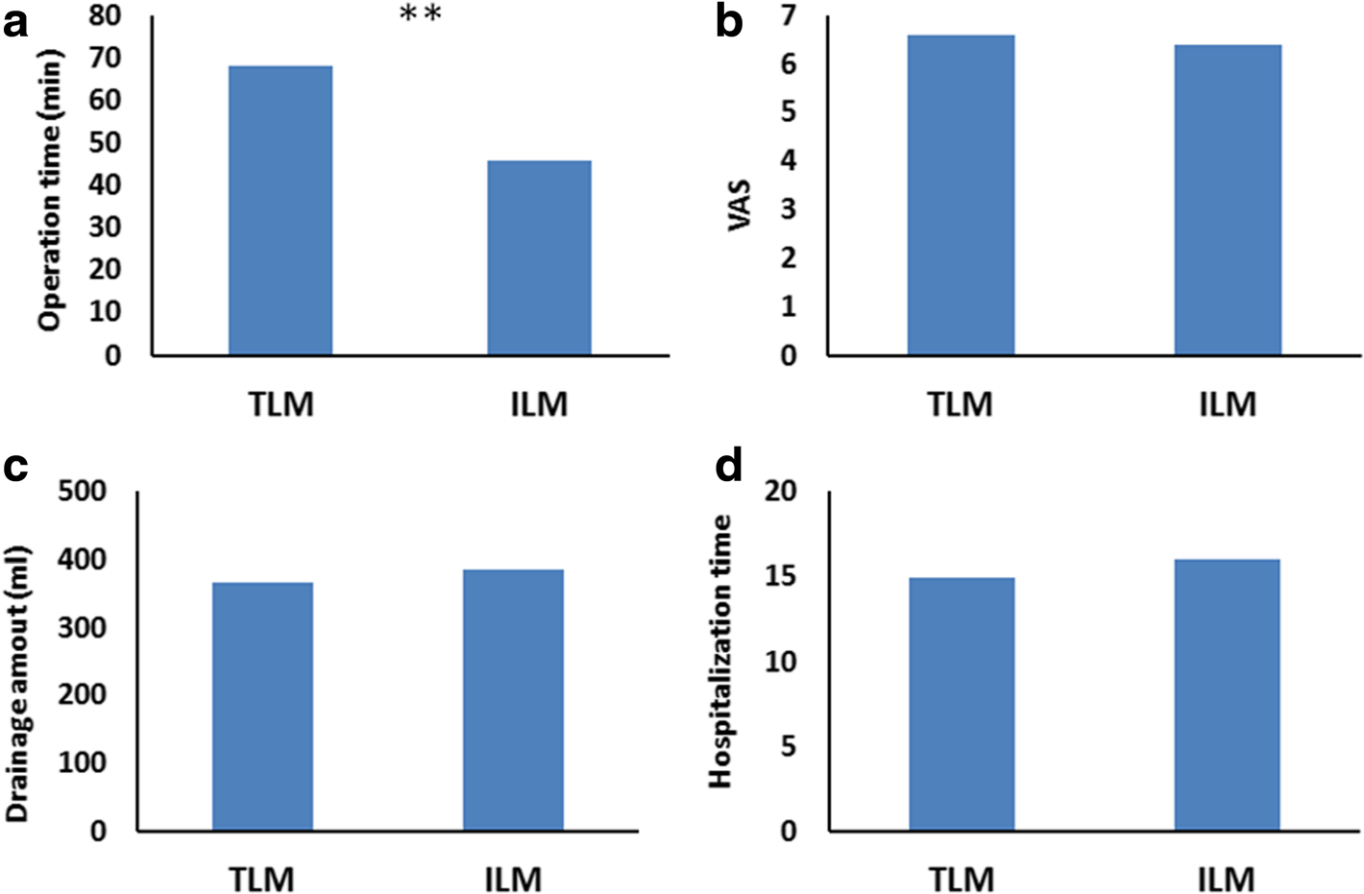
The operation time, VAS, drainage amount, and hospitalization time of TLM and ILM groups are shown in histogram **a**, **b**, **c**, and **d**, respectively. As shown in Fig. 5a, there is significant difference for operation time of the two groups (**<0.05), while for the rest three indexes, no significant difference is confirmed. (*P* > 0.05)

We recorded and calculated four primary measures of the study (Fig. 5). ILM shortened the median EALND time successfully to 46 min, while for TLM group, it was 68 min; the difference was significant (*P* < 0.05) (Fig. 5a). Although ILM significantly shortened the EALND time, it did not compromise the operation outcomes. The median cosmetic results of the two groups were 6.6 and 6.4, the difference was not significant (*P* > 0.05) (Fig. 5b); the median drainage amount of the two groups were 366 and 385 ml (*P* > 0.05) (Fig. 5c); and the median hospitalization time of the two groups are 15 and 16 days; also, no significant difference was confirmed (*P* > 0.05) (Fig. 5d).

The complications of the operations were recorded and calculated too. As shown in Table 2 (complications), the most common complications of ALND such as upper limb paresthesia, reduced arm mobility, postoperative pain, and arm swelling were listed, for our patients received EALND (ILM+TLM), the three rates (3-month postoperation) were 20.3, 15.3, 16.9, and 5.1%.

**Table 2:** Complications

	ILM group	TLM group	Rates (%)
Upper limb paresthesia	7	5	20.3
Reduced arm mobility	3	6	15.3
Postoperative pain	6	4	16.9
Arm swelling	1	2	5.1

## Discussion

Axillary dissection is an important part of the surgical treatment for invasive breast cancer, mostly for prognostic and curative purposes. Because the relatively high complication rate of CALND, during which the injuries to the blood vessels and/or nerves in the axillary fossa were considered to be indicative, and these complications would decrease the quality of life (QOL) of breast cancer patients. In recent years, techniques for early diagnosis and comprehensive treatment for breast cancer have been greatly improved; as a result, the prognosis of these patients has been significantly ameliorated, which means the survival rate and expected lifespan is continuously rising up; thus, the QOL of breast cancer patients has drawn more and more attention. Minimal access surgery/endoscopic techniques could help surgeons to enhance visualization via magnification; the anatomical structure could be clearly observed with the help of the endoscopy, so it could help to decrease tissue trauma, which in turn will decrease postoperation complications. In the 1990s, Salvate [5] and Suzenne [3] reported their work on EALND, which are considered as the beginning of this new procedure used in clinical work for breast cancer. After that, EALND started to be used more and more on breast cancer patients [9]; however, up to today, it is not widely accepted and used, reasons might include the difficulty in learning a new technique and difficulty in adapting to different endoscopic visions, while another important reason might be longer operation time of EALND as compared to CALND [7]. As most surgeons and patients cared a lot about the operation time, especially as Chinese surgeons who need to finish more surgeries compared to our foreign counterparts, we want to find out whether it is possible to shorten the total operation time for EALND.

To carry out EALND, endoscopic operation space (axillary space) has to be established first. In early years, two different methods were used, i.e., the stretching method (dry method) [4, 10] and liposuction method (wet method) [3]. Nowadays, the liposuction method is more popular. For the liposuction method, first the lipolysis liquid would be injected into the axillary fossa and about 20 min [3, 5, 7] would be required before starting the liposuction manipulation. Interestingly, we noticed there are some different formulas for the lipolysis liquid, for example, some researchers used a formula with saline, distilled water, lidocaine, and adrenalin [11, 12], while some other researchers would like to nullify the acidic effect of lidocaine by adding sodium bicarbonate solution [13, 14]. In one of our previous studies, immediate breast reconstruction was tried by combining the EALND technique with laparoscopically harvested pedicled omentum [15]. In this research, we used the same formula and the result was satisfactory.

As mentioned above, after injection of the lipolysis liquid, most researchers [11, 14] would wait 15–20 min before starting liposuction, this is because most surgeons followed the procedure of Salvate [5] and Suzenne [3], they considered this was the time necessary for the fluid to disperse evenly in the axilla. In our study, after the lipolysis liquid was injected, for the ILM group, immediate liposuction was followed, and as shown in Fig. 3a, the axillary space was successfully established, the lens lighting illustrated the space border and it was quite in shape as we planned. Also, we could find the axillary nerves and blood vessels easily and clearly (Figs. 3b and 4a), which meant the adipose tissue was satisfactorily aspirated, almost no any adipose tissue could be found in the operation area. However, in Fig. 4a, we could find some adipose tissues left near the axillary blood vessels, this was because this area was not the target area for axillary lymphadenectomy; we did not inject lipolysis liquid to this level and, during the liposuction procedure, neither did we manipulate in this area.

There were two possible reasons to explain why immediate liposuction could also work. First, as shown in the insets of Fig. 4a, at the beginning of the liposuction, the scratching manipulation of the bi-port cannula was quite important, some adipose tissue particles might be withdrawn directly in this process (white arrow in Fig. 2a). These particles were unable to be observed in the latter part of the liposuction, during which the lipolysis was finished, only oil-like liquid would be found in the aspiration tube (white arrows in Fig. 2b). Venkataram [8] also pointed out that when tumescent liposuction was carried out for aesthetic purpose and under local anesthesia, 30 min should be waited to allow the fluid to percolate uniformly through all layers, while if the liposuction was carried out under general anesthesia, fat could be sucked out just after the fluid was introduced into the fat and cannulae was inserted via incisions, by this way, the process would be much quick. Our breast cancer surgery was carried out under general surgery, we cut off the waiting period in this study, and it proved the liposuction result was satisfactory.

In our study, operation time is set up as a main measure. Many researchers have reported their EALND time, which started with insertion of the optic and ended with skin closure [7]. The operating time of different researchers varied greatly from each other, ranging from about 37 to 168 min [16]. We used about 40–60 min to finish the procedure, which was comparatively shorter to most of the reports, one important reason might be that here in China, the surgeons have more patients to carry out the EALND manipulation; thus, higher efficiency and proficiency might be the result. As reported by Yean et al., the median annual surgeon Medicare volume of breast cancer cases was 6 in their study [17], while for our group, the annual volume was about 200 cases. In fact, such a high volume is also one important drive for us to carry out this study, so as to find the way to minimize the time needed for each operation. As shown in the result, EALND operation time was significantly shorter in the ILM group than in the TLM group (*P* < 0.05). Anyway, such high ranging area of the operation time indicated that internationally standard surgical procedure and training might be necessary.

We also investigated the cosmetic results. Statistical data revealed no significant differences between the two groups for the cosmetic results. Since we only cut off the waiting time for the lipolysis procedure, the rest operation details were exactly the same as each other for the two groups, so it was easy to understand this result. Also, there were no significant differences for the other two measures, i.e., the drainage amount and the hospitalization time of the two groups. We tried to compare the hospitalization time between our study and studies of other researchers but found it was difficult as the discharge standards were quite different among different countries. For example, in some countries where ultra short hospital stay for breast cancer was advocated, patients were discharged from the hospital overnight, which decreased the hospitalization time greatly [18]. However, in our hospital, patients would not be discharged till the drainage tube was removed. Anyway, our data revealed the ILM method did not bring about more drainage amount, and it did not prolong the hospitalization time as compared with the TLM method. The complications of breast cancer surgery, such as the upper limb paresthesia, reduced arm mobility, postoperative pain, and arm swelling were investigated in our study too. As compared with complication rates of CALND previously reported [1, 2], our data indicated complication rates of EALND are lower.

Although liposuction might be used successfully in the endoscopic axillary lymphadenectomy, however, this method does have some limitations. For example, Malur et al. [10] stated that with liposuction, localization of the sentinel lymph node might prove to be difficult, thus this limited usage of liposuction; as we know now, the sentinel lymph node biopsy is considered as a very useful and widely used method, which could avoid axillary lymphadenectomy and thus could reduce postoperation complications for breast cancer patients [19]. The liposuction method is also tiring for the surgeon who needs to remove the adipose tissue with to-and-fro action of the cannulae, some researchers have tried other methods, such as power-assisted liposuction [20] and laser lipolysis [21] for cosmetic purpose, while whether these methods could be used for EALND remained unclear.

It should also be emphasized that the sample volume of our study is limited; also, the follow-up is not long enough (3 months), so more work needs to be done to prove the availability and feasibility of our results.

## Conclusions

Our research suggests that immediate liposuction after tumescent solution injection to the axillary space could shorten the EALND process. At the same time, this method did not compromise the operation results. Although the operation time of EALND, even after our modification, was still longer than CLAND, we advocate the usage of the former, mostly because it could decrease the complication rates of axillary lymphadenectomy for breast cancer patients.

## References

[1] Yeung WM, McPhail SM, Kuys SS (2015). A systematic review of axillary web syndrome (AWS). J Cancer Surviv..

[2] Soares EW, Nagai HM, Bredt LC, da Cunha AD, Andrade RJ, Soares GV (2014). Morbidity after conventional dissection of axillary lymph nodes in breast cancer patients. World J Surg Oncol.

[3] Suzanne F, Emering C, Wattiez A, Bruhat MA (1997). Endoscopic axillary lymphadenectomy after liposuction. Surg Technol Int..

[4] Kamprath S, Bechler J, Kuhne-Heid R, Krause N, Schneider A (1999). Endoscopic axillary lymphadenectomy without prior liposuction. Development of a technique and initial experience. Surg Endosc.

[5] Salvat J, Knopf JF, Ayoubi JM, Slamani L, Vincent-Genod A, Guilbert M (1996). Endoscopic exploration and lymph node sampling of the axilla. Preliminary findings of a randomized pilot study comparing clinical and anatomo-pathologic results of endoscopic axillary lymph node sampling with traditional surgical treatment. Eur J Obstet Gynecol Reprod Biol.

[6] Luo C, Guo W, Yang J, Sun Q, Wei W, Wu S (2012). Comparison of mastoscopic and conventional axillary lymph node dissection in breast cancer: long-term results from a randomized, multicenter trial. Mayo Clin Proc..

[7] de Wilde RL, Schmidt EH, Hesseling M, Mildner R, Frank V, Tenger M (2003). Comparison of classic and endoscopic lymphadenectomy for staging breast cancer. J Am Assoc Gynecol Laparosc..

[8] Venkataram J (2008). Tumescent liposuction: a review. Journal of Cutaneous and Aesthetic Surgery.

[9] Kuhn T, Santjohanser C, Koretz K, Bohm W, Kreienberg R (2000). Axilloscopy and endoscopic sentinel node detection in breast cancer patients. Surg Endosc..

[10] Malur S, Bechler J, Schneider A (2001). Endoscopic axillary lymphadenectomy without prior liposuction in 100 patients with invasive breast cancer. Surg Laparosc Endosc Percutan Tech..

[11] Chengyu L, Jian Z, Xiaoxin J, Hua L, Qi Y, Chen G (2008). Experience of a large series of mastoscopic axillary lymph node dissection. J Surg Oncol..

[12] Chengyu L, Yongqiao Z, Hua L, Xiaoxin J, Chen G, Jing L (2005). A standardized surgical technique for mastoscopic axillary lymph node dissection. Surg Laparosc Endosc Percutan Tech..

[13] Klein JA (1993). Tumescent technique for local anesthesia improves safety in large-volume liposuction. Plast Reconstr Surg..

[14] Brun JL, Rousseau E, Belleannee G, de Mascarel A, Brun G (1998). Axillary lymphadenectomy prepared by fat and lymph node suction in breast cancer. Eur J Surg Oncol..

[15] Zhang P, Luo Y, Deng J, Shao G, Han S, Huang Z. Endoscopic axillary lymphadenectomy combined with laparoscopically harvested pedicled omentum for immediate breast reconstruction. Surg Endosc.29:1376-83 10.1007/s00464-014-3808-z 25159648

[16] Aponte-Rueda ME, Saade Cardenas RA, Saade Aure MJ (2009). Endoscopic axillary dissection: a systematic review of the literature. Breast..

[17] Yen TW, Laud PW, Sparapani RA, Nattinger AB (2014). Surgeon specialization and use of sentinel lymph node biopsy for breast cancer. JAMA Surg..

[18] de Kok M, Frotscher CN, van der Weijden T, Kessels AG, Dirksen CD, van de Velde CJ (2007). Introduction of a breast cancer care programme including ultra short hospital stay in 4 early adopter centres: framework for an implementation study. BMC Cancer..

[19] Lyman GH, Temin S, Edge SB, Newman LA, Turner RR, Weaver DL (2014). Sentinel lymph node biopsy for patients with early-stage breast cancer: American Society of Clinical Oncology clinical practice guideline update. J Clin Oncol..

[20] Coleman WP (2000). Powered liposuction. Dermatol Surg..

[21] Leclere FM, Alcolea JM, Vogt PM, Moreno-Moraga J, Casoli V, Mordon S (2016). Laser-assisted lipolysis for arm contouring in Teimourian grades III and IV: a prospective study involving 22 patients. Plast Surg (Oakv).

